# ‘Good farmers’ and ‘real vets’: social identities, behaviour change and the future of bovine tuberculosis eradication

**DOI:** 10.1186/s13620-023-00245-w

**Published:** 2023-07-27

**Authors:** Gareth Enticott

**Affiliations:** School of Geography and Planning, Cardiff University, Cardiff, CF10 3WA Wales

**Keywords:** Good farming, Bovine tuberculosis, Social research, Farmer behaviour, Identity, Veterinary expertise.

## Abstract

This paper considers the role of social research and human behaviour in attempts to eradicate bTB. Future attempts to eradicate bTB are likely to involve an increasing range of sophisticated technologies. However, the acceptance and use of these technologies is likely to depend on a range of behavioural incentives. The use of appropriate behavioural nudges may facilitate bTB eradication, but the paper contends that of more value are socio-cultural approaches to understanding behaviour. Specifically, the concepts of the ‘good farmer’ and ‘real vets’ are discussed to show how bTB eradication is dependent on social identities. In conclusion, the paper outlines four key roles for social research in assisting with future bTB eradication policies.

## Introduction: social research in the past, present and future of bTB eradication

In seeking to eradicate bovine tuberculosis (bTB), eradication has come to be defined by different practices, in different places, at different times. In the early twentieth century, once eradication had been accepted as a worthy endeavour [1], it became the responsibility of governments to create a disease control infrastructure and impose rules and regulations to reduce the incidence of disease. For countries like the United Kingdom, this approach meant that by 1965, Government could claim to have almost eradicated bTB [2]. In other countries, different approaches have proved more effective. In Australia and New Zealand, the direct influence of government has been replaced by industry owned approaches in which farmers pay and have a say over how eradication should be achieved [3, 4].

The success of these alternative governance arrangements has fed back to countries whose approaches had originally been dependent on state intervention. Countries such as the UK and Ireland, have embraced the discourse of ‘partnership’ in seeking to eradicate bTB [5]. In translating these approaches to new socio-political contexts, however, what has emerged is a hybrid approach, in which the ownership of disease control policy remains problematic, and lacks the success encountered elsewhere [6]. What is different, however, is the emphasis placed upon interventions to promote voluntary behaviour change within farming and veterinary communities, associated with the development of new technologies and practices of diagnosis and prevention.

If the future of bTB eradication rests on the voluntary use of new technologies and practices of eradication, then there is much to learn from both social studies of human behaviour, and the use of technology within agriculture. In this field, attention has recently turned to the so-called fourth agricultural revolution, or ‘agriculture 4.0’ which promises a digital revolution of smart, precise and automated approaches to agricultural practices [7]. Many of these technologies have a direct relationship to the eradication of bTB. Firstly, the creation of big datasets, and online data portals are relevant to both farmers and veterinary epidemiologists. For farmers, these data enable real-time risk-based decisions for cattle purchasing. Similarly, veterinary epidemiologists can use these data to advise farmers and track movements in real-time to trace disease outbreaks. Other technologies use Global Positioning Systems to not only locate cattle, but also control their movements using virtual fencing, set by a farmer on an app and linked to cattle collars to limit their ranging behaviour [8, 9]. With these technologies, farmers could limit the extent to which cattle come into contact with other herds, or graze in areas frequented by wildlife. Finally, new diagnostic tests whose interpretation rests on decision algorithms and machine learning may help to identify animals at risk of testing positive to bTB in future, enabling proactive management by farmers.

Technologies like these promise to make farmers’ lives easier and their businesses more efficient, by enabling more precise and rapid decision making. Nevertheless, analyses of agricultural 4.0 identify a range of concerns including the interoperability and repairability of systems, data security, and the potential loss of labour and expertise [10, 11]. Of particular concern are the way these technologies are developed through top-down processes in which the views of farmers are unaccounted, and/or algorithmic decisions are based on one farming system, and fail to fit others [12]. Indeed, whilst agriculture and disease control’s digital future may foster new opportunities for knowledge exchange, the extent to which farmers engage with and sustainably use these new technologies will be a key focus for future research [13].

Such an agenda requires paying attention not just to these new technologies themselves, but also the increasing reliance on methods of encouraging voluntary behaviour change that accompany them. In this sense, the future of bTB eradication relies less on technological fixes, and more an understanding of the social dynamics of disease control. Whilst financial compensation has acted as a behavioural incentive in many bTB eradication schemes [14], there has been increasing attention paid to the role of a wide range of alternative methods of behaviour change in disease control [15, 16] and which are characterising a new paradigm of bTB eradication.

The remainder of this paper therefore seeks to examine and review evidence on how human behaviour affects the use of disease control technologies and practices; and how future attempts to eradicate bTB might prepare for these challenges given what we know about farmers’ and vets’ behaviour.

## Changing behaviour in animal disease management: from dudge to social identities

Ever since the publication of Thaler and Sunstein’s [17] book, ‘Nudge’, discussions of behaviour change have been dominated by their approach to achieving public goods. Thaler and Sunstein’s approach sits within a paternalist libertarian framework, and proved attractive to governments whose ideology resits regulatory intervention. Drawing on an understanding of mental heuristics and biases in decision making [18], Thaler and Sunstein point to the significance of defaults within choice architecture as a way of scripting and guiding decisions deemed to be in the best interests of individuals or businesses. Thus, the physical layout of a shop, or even paperwork, can be designed to effectively prompt individuals into using or providing the ‘right’ information.

Thaler and Sunstein’s ideas have been incorporated into other behavioural change frameworks often referred to as ‘behavioural insights’. Under these frameworks, interventions designed to change behaviour are encouraged to make decisions easy, attractive, social and timely [19]. For example, carefully placed signs and information may act as cues to make decisions easy; providing information on social norms, i.e. other people’s behaviour in similar geographical or professional contexts can encourage compliance, as can making a decision attractive by linking it to (non)financial incentives (e.g. a reduction in regulation). Some studies have therefore shown how graphical rather than linguistic or numerical information cues may be more effective in changing biosecurity behaviours [16], whilst the provision of information about biosecurity on neighbouring farms prompts a reduction in biosecurity investment [20]. Similarly, experimental research has shown how the provision of bTB risk information in simulated cattle auctions restricts the number of bids, but increases the value of those that do bid [21].

Whilst these ideas have proved attractive to policy makers, others have cautioned against their ubiquity. For some, the ethical dimensions of behavioural change are in danger of being lost in the rush to nudge [22]. Others have suggested that nudge may have proved counterproductive, imagining publics as irrational and infantile [23], achieving only modest gains whilst also directing attention away from systemic challenges that cannot be managed without regulatory intervention [24]. This may be particularly the case where information is relied on to nudge behaviour change, such as in the provision of bTB risk information to guide cattle purchasing. Such initiatives may fail to work because they fail to secure ‘norm internalisation’ providing only a short-term fix [25]. Indeed, other models of behaviour change such as Michie and West’s [26] ‘behaviour change wheel’, suggests that a range of behavioural interventions including both regulatory and persuasive techniques are required to address the range of different behavioural logics ([see for example, [27]).

Approaches to understanding behaviour through social and cultural identities provide a different critique of the nudge philosophy. According to social identity theory, failures of nudge techniques and the provision of information to prompt behaviour change are particularly evident when these techniques are applied to issues that require collective action to manage risks, such as disease control [28]. Existential threats to communities can lead to the mobilisation and coordination of collective solutions to ensure the community as a whole benefits rather than just the most able [29]. The implications of these critiques for cattle purchasing is that behavioural change interventions may be most effective when they are designed and produced by the communities affected by them [30]. At the same time, the social identity approach emphasises the importance of other factors such as leadership that can cultivate a feeling of ‘us-ness’ around which communities can rally behind [28]. Indeed, accounts of successful bTB eradication in Australia clearly speak to the importance of leadership and clear, shared collective aims and benefits [3].

### ‘Good Farmers’ and ‘Real Vets’

Helpfully, in social studies of agriculture and veterinary practice there are already conceptual frameworks that build on these social identity approaches to behaviour change. These frameworks situate understandings of farmer and vet behaviour within their socio-cultural environment, providing a guide to how their behaviour can be understood, and the challenges to and conditions by which behaviours may be changed.

In social studies of farming, the concepts of ‘good farming’ and the ‘good farmer’ have been used to understand farmers’ behaviour and identify methods of changing behaviour that rely on the significance of socio-cultural identities. Developed by Burton and others ([see [31]), the concept of good farming stems primarily from a primary need to have a clear sense of collective identity and group belonging. Group identities exist around a core set of shared principles – known as the ‘rules of the game’. These rules are learned through socialisation that form familiar stories which literally script behaviours and express a cultural groups’ wider values. In farming studies, often these shared rules are the importance of ‘hard’ or ‘real’ work; the importance of being clean and tidy; and being part of the community or a good neighbour. The rules of the game are also articulated symbolically which allows people to visually demonstrate their group allegiance and others to recognise who is in and who is out. Symbols encode what is valuable forming what is called cultural capital. The more cultural capital, the more status one has as a good farmer and the more one is trusted by others in farming.

Examples of the visible symbols of good farming include a ploughed field. It is highly symbolic – if it is straight – because it demonstrates the practical skills of farming, whilst the crops that grow in the field – if they look healthy and productive – will be symbolic of these skills too. New machinery, whilst reflecting farmers’ economic capital, may also symbolise cultural values of hard work. Importantly, all of this is visible to other farmers driving past the farm, engaging in what is referred to as ‘roadside’ or ‘hedgerow farming’ – the practice of surveillance and evaluation of which other local farmers can be seen to be good farmers [31].

By contrast, work that is conducted indoors – paperwork and bureaucracy is not valued because it holds limited symbolic value: it is not visible, it is not real work, and it doesn’t rely on practical skills of farming. The failure of these aspects of work to integrate within agricultural identities explains why voluntary reforms relating to, for example, agri-environmental schemes, are unpopular. RJF Burton [32] argues that where schemes promote activities that do not reflect these cultural identities, even if they provide financial rewards, they fail to promote widespread cultural change, and just reward those farmers who had already adopted these practices. Thus, RJF Burton and UH Paragahawewa [33] encourage policy makers to think about how policies may provide symbolic rewards that contribute to the good farming identity.

Studies of animal disease management have identified similar connections between good farming symbols and disease management practices, such as the importance of cleanliness and good-looking stock (see [34, 35]). The extent to which the control of bTB can be tied to symbolic aspects of identity is less clear, however. Studies have shown for instance that whilst there are symbolic dimensions of good farming that influence cattle purchasing [36], attempts to link disease management to good farming can be undermined by farmers’ own understandings of disease transmission [37]. This is explored in more detail in Sect. 4.

Much the same ideas can be applied to the veterinary profession. All professions have a set of competing identities based around their practices and values, which is encapsulated within a ‘master narrative’ [38]. The master narrative prescribes the limits of professional identity, regulates professional behaviour and determines professional status: in short it defines who is a ‘real vet’. The master narrative therefore serves to reinforce dominant professional identities: those on the margins are not recognised as legitimate or valuable, are less able to intervene or voice concerns. Master narratives may be created within professional, education and policy institutions that define the ‘ideal type’ of worker. Equally, the master narrative may be created and reinforced by the media, as well as through day-to-day professional work itself.

In the UK, the most powerful example of this is the James Herriot books and TV programmes, shaping how prospective vets imagine the day-to-day existence of a farm animal vet. The valourisation of these parts of the profession is reflected in how vets themselves see their colleagues. Agathou et al’s [39] analysis of positive and negative sentiments used by vets when describing different specialities within the veterinary profession shows how farm vets are thought of positively through the terms strong and practical. Epidemiologists, by contrast, are dismissed as ‘nerdy’ and ‘boring’. Even though both may deal with disease, the distinctions between them may be related to the symbolic dimensions of where and how they do their work: do they work in an office hunched over computers dealing with vast datasets; or are they out in the field, talking to farmers and touching live animals?

These symbolic dimensions of veterinary practice speak not just to other vets, but to their clients (such as farmers) too. Vets who regularly share the same work and social spaces as farmers, and display symbols of identity demonstrate what is referred to as ‘bonding’ and ‘bridging’ capital – the ability to cross professional and cultural boundaries and become trusted advisors (see [40]). In this way, these forms of cultural capital allow vets to create a joint sense of collective identity with farmers and in doing so become recognised as ‘real vets’. As demonstrated in the following section, this can have important implications for attempts to eradicate bTB. At the same time, how vets are enrolled into the bTB eradication attempts may dampen or enthuse their participation. Where these attempts do not reflect the symbolic identity of the ‘real vet’, it would be no surprise to see vets suffer from what PJ Burke [41] refers to as ‘status anxiety’ in which veterinary work fails to reflect its representation in the master narrative. Endless rounds of bTB testing may not correspond with what a real vet should be doing resulting in not just a failure to engage with the management of bTB, but exit from the profession altogether [42].

## Illustrating the ‘real vets’ and ‘good farmer’ in bTB eradication

To illustrate how socio-cultural perspectives of good farming and the real vet can help us understand and shape approaches to bTB eradicate, this section draws briefly on two case studies. The first relates to the role of the veterinary profession in bTB eradication (for more details see [43]); and the second relates to farmers’ cattle purchasing practices (for more details see [36]). In common to both is the importance of cultural capital and trust in guiding behaviour in bTB eradication, but both also point to the behavioural components of eradication schemes in future.

### 'Real vets' and the eradication of bTB in New Zealand

Contemporary bTB eradication attempts in New Zealand have been lauded as a successful example of industry ownership of disease management. Yet the journey to eradication has not always been plain sailing, riven with conflict and at times a ‘toxic’ relationship between the Government, farmers and between different sections of the veterinary profession. Learning from these past experiences can be instructive, for what they reveal is how conflicts over who or what is a ‘real vet’ became central to the success of eradication attempts in New Zealand.

The architect of New Zealand’s first attempt at bTB eradication was a Scottish vet, Dr Sam Jamieson. He had emigrated to New Zealand in 1952, to escape post-war Britain and determined to live a quiet life devoting as much time as possible to fishing. Jamieson had experience of bTB from his work as a lecturer at Aberdeen University in Scotland, and arrived in New Zealand in 1952 to work as a veterinarian to supervise the Meat Works, sign export certificates and supervise bTB testing as part of the tuberculin testing scheme in milk supply herds which had begun in 1951. Jamieson recognised the testing programme was not functioning and in 1953, reluctantly, attended a meeting about the future of the scheme. From here, Jamieson became one of New Zealand’s most influential vets: acting as a technical advisor to the 1954-6 Government inquiry into bTB control; designing and implementing the bTB eradication plan that began in 1961; and becoming the first Director of the Animal Health Division in the Department of Agriculture.

For Jamieson, veterinary work involved setting and following rules. Action was directed from the centre of government. Only government vets could advise on TB as only they had sufficient knowledge. His approach to eradication was pretty simple: what he said went. According to his obituary, Jamieson’s style was ‘autocratic’. Farmers needed to be taught and educated about TB and his communication was blunt. Colleagues recalled his straight talking and no-nonsense stand made him plenty of enemies. Jamieson agreed with this characterisation, noting: ‘*I don’t push people around and I’m not aggressive…But people who come to me should know their responsibilities as well as I know mine. I don’t make any bones about how I feel toward people who have a half-knowledge and try to get involved in my field’.*

Jamieson expected other vets to fall into line. Most did, but one who didn’t was a vet called Peter Malone based in Nelson. The dispute that emerged between the two characterises distinctions between different forms of veterinary conduct, and the kind of relationships with farmers that do or don’t make eradication work. In the early stages of eradication in New Zealand, the prevalence of TB was such that farmers were losing cattle to such an extent that it threatened their business. Their concern was that the test was not working, and it was Malone’s too. Malone took it upon himself to investigate: he would ‘read light’ as he put it, giving the benefit of the doubt to younger stock which he noticed subsequently tested negative. Compared to Jamieson’s universal rule-bound approach to veterinary work, Malone’s was more individual and flexible and accommodative of local environmental conditions connected to the problem of non-specificity.

Malone let this slip in a meeting with the Ministry one day: when Jamieson found out he was outraged. Malone was suspended from all bTB eradication activities. If Jamieson thought that was the end of the matter he was wrong. Farmers were also outraged. They went on strike and refused to test: they had no trust in the programme. The Ministry dispatched one of their veterinary staff to find out what was going on. His conclusion below bears witness to the importance of the cultural capital of ‘real vets’ in establishing and maintaining trusting relationships with farmers:

[Farmers] feel they have been neglected, exploited and forgotten over the years. They are convinced that they cannot expect help and understanding from the central government…and this persecution complex has been strengthened further by the knowledge that the one man who has helped them, and tried to bring their difficulties over high losses from TB testing to the attention of Wellington, has been penalised by having his licence cancelled

As a way of diffusing this dispute, Jamieson organised a trial of diagnostics to settle the arguments with science. Public demonstrations of the reliability of bTB diagnostics had already been used by Jamieson in which trained vets correctly diagnosed cattle with bTB. This time, Jamieson organised a diagnostic trial which involved railroading hundreds of cattle to at an agricultural college called Flock House before subjecting them to a range of bTB tests, and conducting post-mortems. The scientific results of the trial allowed Jamieson to say that the tests did a good job, but farming organisations took a different view, pointing to the number of false positives that the tests found.

This controversy therefore reveals the distinctions between different sectors of the veterinary profession in disease management. For farmers, veterinary status was not revealed by scientific practices and processes. Rather, trust was located in personal social relations, in which vets demonstrated an ability to care for and understand local environments. These were the real vets for farmers, those possessing the ability to take farmers with them and understand them, rather than trying to rule from a distance. Thus, this story shows the importance of the social relations of disease eradication: who is involved in veterinary practices, and how farmers are engaged in disease eradication.

### 'Good farming' and the eradication of bTB in England

If good vets are important to the progress of bTB eradication, so too are good farmers. This is particularly the case when it comes to cattle purchasing. Providing farmers with more information about the disease status of cattle as a way of encouraging risk-based trading has become a key part of bTB eradication attempts to prevent the movement of risky stock between areas of high and low bTB prevalence. Providing this information repeats the mistakes of the deficit approach to risk communication, assuming that information provides the key to decision making: the ‘right’ information will lead to the ‘right’ decision. To be sure, this approach will be relevant to some farmers, who are data hungry, but in general research [44] identifies a broad typology of farmers with different approaches to cattle purchasing:

i) **The Chancer** - purchases are motivated by ‘good value’ judged using intuitive skills to identify what appears to be a ‘bargain’, and mostly purchased at markets. Chancers may buy cattle on a whim that are not immediately needed, or their purchases may address short-term needs such as the urgent need for a bull or a calf.
ii) **The Entrepreneur** – purchases are driven by financial margins. They are risk takers and have much in common with the chancer: all purchases are seen as gambles with inherent risks, but each is weighed up on a financial basis and taken where they make sense through an economic lens. Entrepreneurs may be new entrants, forced into adopting a hard-nosed approach to cattle purchasing by their lack of capital. This means they may care less about what the animal looks like, but what they can make from it.
iii) **The Manager** – these farmers carefully weigh up purchasing decisions, buying cattle to match existing priorities and contractual requirements. Data is important to these purchasing: milk yields and health status will be considered by these farmers. Beef farmers will pay attention to other statistics such as EBVs and calving ease. Purchases may be from dispersal sales or directly from known good farmers to provide reassurances over the quality and planned in advance.
iv) **The Stockman** – purchases are long term investments. The stockman is interested in detailed data of the herd, farmer and individual animals that they buy. Their choice of cattle may be down to personal choice: they are likely to have their own breed preferences and be invested in the long-term genetic improvement of breeds. Shape and size of animals they buy is very important, and animals are not expected to fit a system more that the system cares for those animals. In general, all purchases are carefully planned and considered, but will also rely on personal connections and knowledge.
v) **The Professional/Traders** - their skill lies in the use of the stockman’s eye to spot ‘good value’ but also to match cattle to specific systems. They have a sharp eye for detail and an awareness of the market, and a wide range of contacts and understanding of different farming systems. In the market, they are in direct competition with entrepreneurs who may view them with suspicion and question their value to farming.

For each of these segments, purchasing strategies aim to buy cattle that fit each farm’s system. ‘Fitting the system’ requires cattle to fit in with the natural and physical environment, and the economic and social environment, including labour and family commitments. The system is therefore highly complex and determines how decisions are made (for an example of this complexity in Northern Ireland, see [45]). Farmers can help to preserve the system working by choosing the right cattle (for an example of this in New Zealand, see [46]), but systems can be disrupted leading to new farming practices. Indeed, when thinking about the triggers that prompt changes to the way cattle are purchased, research suggests that this is mediated by significant disruptions to the farm system, and which can make some forms of information and advice more or less salient. Thus, a recent big breakdown may mean that risk information is more important; or prioritise productive cattle to meet a milk processor’s contractual requirements. But just as important can be family reasons – unexpected deaths, succession, births and marriages all create windows of opportunity in which purchasing practices can suddenly change. Succession may allow a new farmer to implement ideas they have had for many years but have been unable to implement. A young family may lead to the farm being reconfigured to make life easier, challenging the traditional ‘hard work’ of the good farmer.

As this cattle purchasing segmentation shows, in fitting the system, farmers will rely on different forms of information to make their choices, and whilst objective information on disease risks may be important for some, the intuitive skills of the good farmer to recognize by sight a ‘good cow’ will also be relevant. Reliance on these skills will be particularly relevant when the marketplace is imperfect with poor availability and information which conflicts with other important criteria. In the face of all this uncertainty and complexity, it’s not a surprise to see farmers relying on what is familiar to them when making decisions. Strategies are developed to balance different qualities – and for cattle purchasing, an important strategy is knowing whether the vendor can be trusted: are they in short a ‘good farmer’?

At the market, farmers will look to purchase from “genuine sellers” – farmers who possess the kinds of symbolic capital that distinguishes who is a ‘good farmer’. Farmers buy ‘the man and the beast’ and seek assurance in the visible qualities of a good farmer: clean and tidy stock; and farmyards that are free from ‘junk’ and plastic waste littering the farm. At some markets, there are some practices that can help discern who is trustworthy: ‘standing behind your animals’ in the auction box at markets can offer reassurance that the vendor is genuine. Other traditions may also symbolise trust. In some parts of England, “Luck money” is offered by the vendor during the sale – it is visibly held up by the seller during bidding. Its only a small amount (for example, a £10 note) but symbolises much more – the legitimacy of the seller and commitment to the agricultural community.

The significance of these symbolic displays of ‘good farming’ is such that they can override, or at the very least mediate, objective risk data. In doing so, they can demonstrate the disconnect between ‘good farming’ and disease management. This is why farmers will go back to the same supplier after they have had a TB breakdown – because their trust is based on other symbolic capital. TB may have just been bad luck. Similarly, it is why dispersal sales will be well attended: out of respect for and a commitment to being part of the farming and wider rural community. Turning up is as much a statement of one own’s good farming credentials as it as recognition of the farmer who is selling.

## Conclusion: how to use social research to eradicate bTB

As suggested at the start of this paper, future attempts to eradicate bTB are likely to be dependent on new technologies that better predict and eliminate disease. Whilst these technologies will be led by veterinary science, I contend that social research will also play an increasingly important role. This is because, as we I have shown historically in relation to bTB testing, and more recently in relation to cattle purchasing, technologies and practices of disease eradication are mediated by socio-cultural processes. To avoid repeating the mistakes of the past and the present, future eradication attempts need to learn from these lessons. In conclusion, I offer four ways in which social research may help with this process.

Firstly, social research highlights the lived reality of disease eradication for farmers and vets. Various studies have revealed the social and economic costs of disease eradication, for bTB and other diseases [47–49]. These and other studies of the impact of disease control policies are important because they describe the social contexts in which disease eradication attempts operate, the complexity of decisions facing farmers, and the limits to eradication. Understanding the social context of disease eradication is crucial in designing effective disease control policies, establishing expected outcomes and finding the balance between different regulatory approaches. Thus, whilst social research may temper our expectations about how eradication can be achieved (if at all), it can nevertheless provide guidance on how behavioural changes amongst farmers may be best achieved.

Secondly, the perception of what is a ‘real vet’ and who can be trusted is central to the acceptance and development of diagnostics. The distinctions between different forms of veterinary expertise involved in disease eradication highlights the importance of those forms of cultural capital that allow vets to work across cultural and professional boundaries. In highlighting these distinctions through the account of early bTB eradication in New Zealand, the point has not been to suggest which forms of veterinary expertise are best, i.e. who are the real vets, and how can we get more of them. Rather, the distinctions that are made between ‘real vets’ highlight the importance of the social organisation of disease control. Indeed, what emerged in New Zealand following the dispute between Malone and Jamieson was a more consensual kind of veterinary expertise which sought to cross divides, combining veterinary expertise with farmers’ own expertise. Social research therefore alerts us to the importance of developing new organisational structures which can seek to improve trust between scientists, vets and farmers.

Thirdly, social research impresses on us how farmers are not all the same and can help us be smarter about how we seek to change farmers’ behaviour. Consumer marketing invests great time and money to segment and then target specific groups of consumers with specific messages at particular times of their lives. We know that farmers may be open to certain ideas and messages at some times of the year, and at some times of their lives. A smart TB eradication programme would therefore seek to know much more about the people it was trying to persuade to behave differently and organise its communication accordingly.

Finally, social research shows how farmers’ behaviours are linked to their socio-cultural identities, or more specifically their attachment to what they consider to be good farming. The reliance on good farming as a decision tool speaks to the way we all seek familiarity in the face of complexity. If farmers make decisions contrary to veterinary guidance, it does not mean that they are bad farmers, just that their decision-making processes are guided by a different set of instruments. The importance of understanding social identities contributes to the first point, but it also speaks to the power of ‘us’ – the importance of shared goals, common identities, and inclusive leadership. One consequence of this could be to re-imagine bTB eradication in relation to the benefits it provides to farmers’ own social identities rather than framing it as a complex technical task. Indeed, given the politicisation of bTB eradication in some countries like Ireland and the UK, it may be that more can be achieved by not even referring to bTB, given that many bTB biosecurity interventions are standard farming practices. This kind of depoliticization would allow disease control practices to be firmly related to farmers’ own sense of what is good farming and could rely on those people who are trusted by farmers as ‘real vets’.

## Data Availability

Data are not available.

## Abbreviations

bTB: bovine tuberculosis
